# Evidence that seasonal malaria chemoprevention with SPAQ influences blood and pre-erythrocytic stage antibody responses of *Plasmodium falciparum* infections in Niger

**DOI:** 10.1186/s12936-020-03550-9

**Published:** 2021-01-01

**Authors:** Lamine Mahaman Moustapha, Rafiou Adamou, Maman Laminou Ibrahim, Mariama Abdoulaye Louis Padounou, Abdoulaye Diallo, David Courtin, Jean Testa, Jean Louis Abdourahim Ndiaye

**Affiliations:** 1 Université Cheick Anta Diop-Dakar, Dakar, Sénégal; 2 Centre de Recherche Médicale et Sanitaire-Niamey, Niamey, Niger; 3 Laboratoire de Biochimie et de Biologie Moléculaire, Faculté des Sciences et Techniques, Université d’Abomey-Calavi, Abomey Calavi, Bénin; 4 MERIT UMR 216, Institut de Recherche pour le Développement, Université Paris Descartes, COMUE Sorbonne Paris Cité, Paris, 75006 France

**Keywords:** Seasonal malaria chemoprevention, Immunity, Antibody, CSP, GLURP-R2, *P. falciparum*

## Abstract

**Background:**

In endemic areas, children develop slowly and naturally anti-*Plasmodium* antibodies and become semi-immune. Seasonal Malaria Chemoprevention (SMC) with sulfadoxine-pyrimethamine + amodiaquine (SPAQ) is a new strategy to reduce malaria morbidity in West African young children. However, SMC may impact on the natural acquisition of anti-*Plasmodium* immunity. This paper evaluates the effect of SMC with SPAQ on antibody concentration in young children from Niger.

**Methods:**

This research was conducted in areas benefitting from SMC since 2014 (Zinder district), without SMC (Dosso district), and with 1 year of SMC since 2016 (Gaya district). To assess the relationship between SMC and *Plasmodium falciparum* IgG antibody responses, the total antibody concentrations against two *P. falciparum* asexual stage vaccine candidate antigens, circumsporozoite protein (CSP) and glutamate-rich protein R2 (GLURP-R2), in children aged 3 to 59 months across the three areas were compared. Antibody concentrations are quantified using an enzyme-linked immunosorbent assay on the elution extracted from positive and negative malaria Rapid Diagnostic Test cassettes.

**Results:**

The analysis concerns two hundred and twenty-nine children aged from 3 to 59 months: 71 in Zinder, 77 in Dosso, and 81 in Gaya. In Zinder (CSP = 17.5 µg/ml and GLURP-R2 = 14.3 µg/ml) median antibody concentration observed are higher than in Gaya (CSP = 7.7 µg/ml and GLURP-R2 = 6.5 µg/ml) and Dosso (CSP = 4.5 µg/ml and GLURP-R2 = 3.6 µg/ml) (p < 0.0001).

**Conclusion:**

The research reveals some evidences which show that seasonal malaria chemoprevention with SPAQ has an effect on blood stage antibody responses and pre-erythrocytic stage of *P. falciparum* infections in Niger. Increased antibody titres with increased SMC/SPAQ implementation. This contradicts hypothesis that SMC/SPAQ could reduce immunity to erythrocyte and liver-stage antigens. Further studies are necessary to provide better understanding of the SMC effect on malaria immunity.

## Background

Malaria caused by *Plasmodium falciparum* remains the major cause of morbidity and mortality in children under 5 years in sub-Saharan Africa [1]. It is the main public health problem in Niger [2]. The national malaria control programme of Niger has implemented complementary malaria control strategies based on World Health Organization (WHO) recommendations, including seasonal malaria chemoprevention (SMC) with sulfadoxine-pyrimethamine + amodiaquine (SPAQ) [3–5]. SMC is an administration of full therapeutic doses of these drugs to children aged 3 to 59 months at monthly intervals during malaria season in endemic areas [6]. Without SMC interventions, children slowly develop anti-malarial antibodies, naturally becoming semi-immune [7, 8].

Sulfadoxine is an antibacterial and anti-malarial drug of the chemical class of sulfonamides. It is a dihydropteroate synthetase (dhps) inhibitor, a key enzyme in the biosynthesis of folate. It acts by competitive inhibition of para amino benzoic acid (PABA) to block the synthesis of folic acid and *Plasmodium* nucleotides (purines and pyrimidines). Pyrimethamine associated with sulfadoxine (SP) is a competitive inhibitor of dihydrofolate reductase (dhfr), a key enzyme in the redox cycle for the production of tetrahydrofolate, a cofactor necessary for the biosynthesis of DNA and proteins. SP acts on the asexual forms of the hepatic and erythrocytic stage of *Plasmodium*.

Amodiaquine is an anti-malarial with antipyretic and anti-inflammatory properties. It is a 4-aminoquinoline related to structure and activity with chloroquine. Amodiaquine is active on the erythrocyte form of *Plasmodium*.

Malaria immunity is partial, short-lived, and requires exposure to infected mosquitoes bites to be maintained [7]. Monthly given SMC reduces malaria morbidity in West African children [5, 9–11]. However, SMC may impact on the natural acquisition of anti-*Plasmodium* immunity. In Senegal, Ndiaye et al. suggest that long-term SMC by SPAQ has limited impact on the development of acquired immunity [12]. In the same country, Sylla et al. show that SMC with SPAQ can induce the decrease of IgG anti-AMA1 and anti-MSP1__42_ [13]. In Mali, Mahamar et al. conclude that exposure to SMC/SPAQ lowers anti-AMA-1, MSP1__42_ and CSP titers [14]. Other Malian studies maintain that the duration of exposure to SMC had no effect on antibody to MSP1__42_ and CSP [14].

The hypothesis of this study is that SMC/SPAQ could reduce immunity to erythrocyte stage antigens and liver-stage, and malaria Rapid Diagnostic Tests (RDT) absorption filters could be used to measure IgG titers. To assess the relationship between SMC and *P. falciparum* antibody responses, the total IgG concentrations against two *P. falciparum* asexual stage vaccine candidate antigens: circumsporozoite protein (CSP) and glutamate-rich protein R2 (GLURP-R2), of children aged 3 to 59 months across the three sites were compared. The CSP is a secreted antigen of the pre-erythrocyte stage of *Plasmodium* and GLURP-R2 is an antigen associated with mature schizont of blood stage. Antibody concentrations are quantified using an enzyme-linked immunosorbent assay (ELISA) on the elution extracted from positive and negative RDT cassettes.

## Methods

### Study design and sample collection

The data presented here are generated from the malaria morbidity sentinel surveillance sites within the SMC programme in Zinder, Dosso and Gaya districts located in western Niger, where malaria transmission is seasonal [15, 16]. Zinder and Gaya districts have implemented SMC with SPAQ, respectively since 2014 and 2016; they are classified as meso-endemic and hyperendemic areas [15, 16]. SMC is not implemented in Dosso district, which is classified as hyperendemic and as a control district of the study [15, 16].

In 2016, the reports on SMC Coverage from Zinder showed the following results:

- Report #1: 91%.
- Report #2: 73%.
- Report #3: 50% (Unpublished data)..

The coverage of Gaya district were 77.72%, 81.56%, 71.26%, and 69.47 respectively for round 1, 2, 3 and 4 (Unpublished data). All the 3 sites used Artemisinin-based Combination Therapy (ACT) as first line treatment for uncomplicated malaria cases and all received universal coverage of bed nets. The seasonality of malaria transmission in these 3 sites is the same.

To assess the impact of SMC on the titer of antibodies to two asexual *P. falciparum* stage antigens, 6 health facilities in Zinder, Dosso and Gaya were selected. In these health facilities, malaria RDTs (SD-Bioline) of randomly selected children aged 3 to 59 months are collected from symptomatic cases (fever + positive or negative RDTs) for serological analysis. For all RDTs collected, the date of consultation, the age, the gender, whether the RDT test was performed and the result of RDT test (positive or negative were reported on the cassette. Samples were collected all three months at the same time in all sites between November 2015 and December 2016. The RDTs were stored at room temperature. The analyses were performed in April 2017. The average periods of the RDT before testing across the sites were: 06 month for Zinder, 05 month for Dosso and 7 month for Gaya.

The total number of children concerned was 249, and the computation based on 78% circumsporozoite protein antibody prevalence in children that received SMC during 1 to 3 years [14] (95% CI) with a precision of 5%.

### Recombinant antigens

The malaria antigens used in this study include a recombinant antigen circumsporozoite protein (CSP) and glutamate-rich protein R2 (GLURP-R2). CSP antigen is a 44-aa NANP repeat-sequence peptide of the *P. falciparum* circumsporozite protein synthesized by Sygma Genosys, while GLURP-R2 (amino acids 706-1178, F32 strain) is an amino acid produced by the Statens Serum Institutes of Copenhagen (Denmark) and is expressed in *Escherichia coli*.

### Serum elution

Serum is eluted from filter paper inside RDTs cassette collected [17]. RDTs have proximal, middle and distal parts according to the description by Cnops et al. [18]. The distal part of RDT contains a filter paper component that absorbs the residual blood solution. The cassettes are opened by sterile tweezers and distal part of each RDT is cut with sterile scissors in two or three pieces about 2 mm and eluted (all pieces obtained) into 300 µl of phosphate buffered saline (PBS) from this fragment placed in 1.5 ml Eppendorf tubes. The solution is stored at 4 °C overnight. It is equivalent to a 1:100 dilution of whole blood, which is approximately 1:200 with respect to serum or plasma, assuming 50% haematocrit [17]. The elutions CSP and GLURP-R2 total IgG antibody responses are quantified using ELISA [19].

### Antibody measurements

The standard operating procedure developed by the African Malaria Network Trust was used to assess total IgG concentrations by ELISA to CSP and GLURP R2, as described previously [20]. Briefly, recombinant proteins (0.1 µg/well) diluted in PBS were coated on MaxiSorp Nunc plates (Thermo Fisher Scientific, Denmark) and blocked with 3% powdered-milk + 0.1% of PBS-Tween 20. Sera samples were diluted at 1:200 for all recombinant proteins. Polyclonal goat anti-human IgG (Gamma) (Caltag) conjugated to HRPO diluted 1:3000 (Skybio, France) was used for revealing the reaction with 3,3′,5,5′-tetramethylbenzidine TMB as substrate and 0.2 M H_2_SO_4_ to stop the reaction. Standard curves were established using human IgG purified proteins (Binding Site, France) to determine the concentration of specific antibodies. Concentrations of the standard curve are as follows: 500, 250, 125, 62.5, 31.3, 15.6, 7.8, 151 and 3.9 µg/mL. Each curve is run in duplicate on each plate. The ADAMSEL FLP b039 software [21] is used to analyse absorbance at 450 nm and interpolate the standard curve (µg/ml). Discordant duplicates (with a variation coefficient > 15%) are dropped.

### Statistical analysis

The Median test is used to analyse differences between IgG medians concentrations. The comparisons between IgG median concentrations in Zinder, Dosso and Gaya are performed to investigate the potential impact of SMC on antibody responses. Positive and negative cassette results as well as results between districts were compared. The comparison between IgG median concentrations are performed by Mann–Whitney test. Data are analysed with SPSS software version 16.0. P-values ≤ 0.05 are considered statistically significant.

## Results

### Population characteristics

**Fig. 1:**
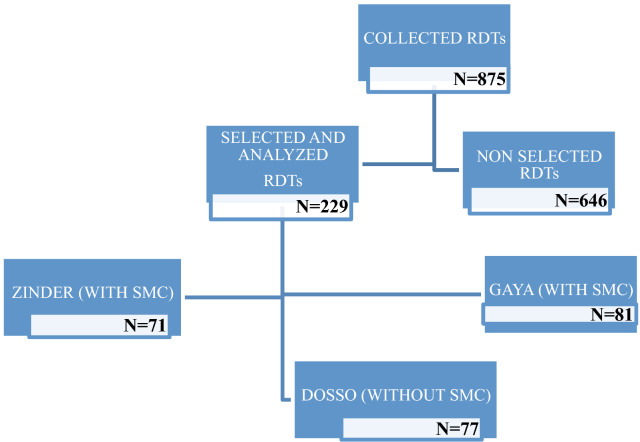
Characteristics of samples collected. A total of 875 RDTs were collected, of which 229 were analysed and 646 were not analysed. Of 229 included in the analysis 7 were from Zinder, 77 from Dosso, and 81 from Gaya

A total of 229 children aged 3 to 59 months were concerned by the analysis: 71 from Zinder, 77 from Dosso, and 81 from Gaya (Fig. 1). The number of samples categorized by mean age and gender is comparable between districts and differences are seen between RDT results (Table 1). Using ANOVA test no statistical difference of mean age between the sites is showed (p = 0.11).

**Table 1:** Characteristics of children included in the survey

Characteristic	Zinder (N = 71)	Dosso (N = 77)	Gaya (N = 81)
Age (months)			
Mean age	30.2 ± 17.5	25.4 ± 14.5	25.0 ± 15.4
Sex (%)			
M	53	50	63
F	47	50	37
RDTs results (%)			
Positive	60.3	66.2	44.44
Negative	39.7	33.8	55.56

Zinder district (SMC since 2014), Dosso district (No SMC) and Gaya district (SMC 2016)

### Anti-CSP and GLURP-R2 IgG median antibody concentrations by districts

**Fig. 2:**
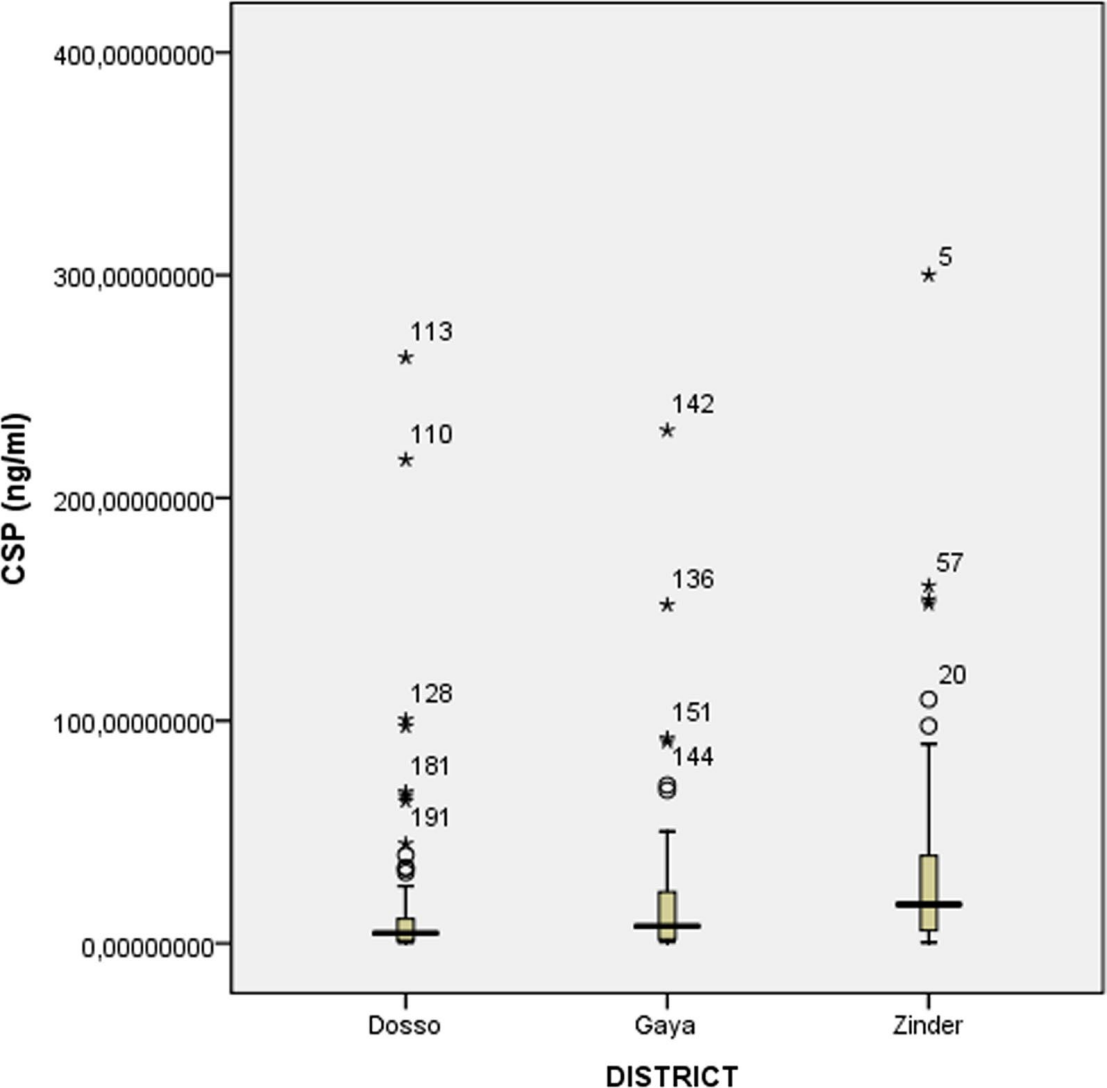
Comparison of anti-CSP IgG antibody median concentrations. Zinder district (SMC since 2014), Dosso district (No SMC) and Gaya district (SMC since 2016). Comparison of the three districts simultaneous is performed using a Median test and two by two with Mann–Whitney test. The significance limit was p < 0.05

**Fig. 3:**
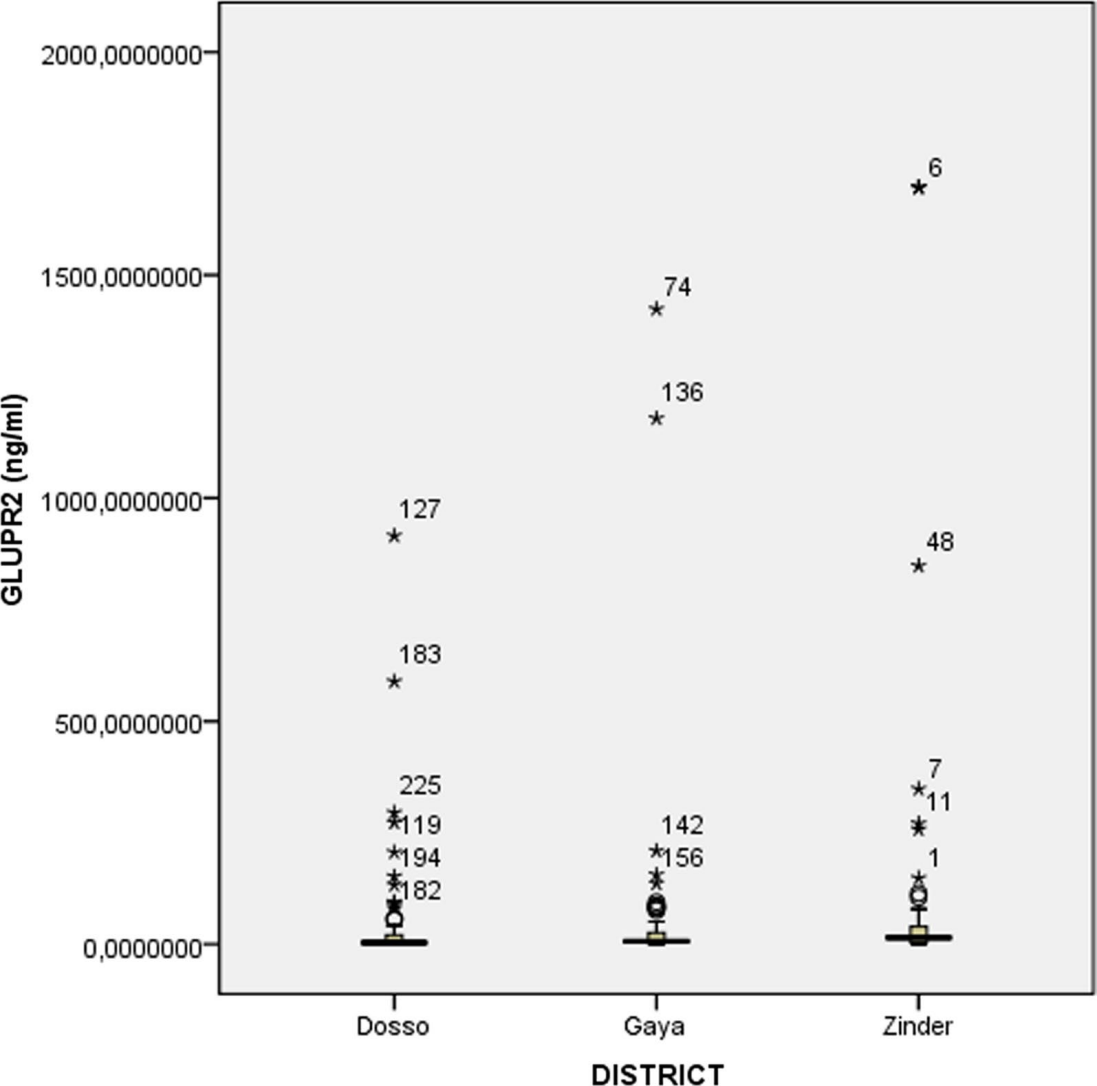
Comparison of anti-GLURP-R2 IgG antibody median concentrations. Zinder district (SMC since 2014), Dosso district (No SMC) and Gaya district (SMC since 2016). Comparison of the three districts simultaneous is performed using a Median test and two by two with Mann–Whitney test. The significance limit was p < 0.05

In Zinder with SMC since 2014 (CSP = 17.5 µg/ml and GLURP-R2 = 14.3 µg/ml) and Dosso with no SMC (CSP = 4.5 µg/ml and GLURP-R2 = 3.6 µg/ml), median concentration of IgG antibody responses has been significantly different because of the two antigens by districts (Figs. 2 and 3). No significant difference in median concentration of all antibodies is shown between Dosso (CSP = 4.5 µg/ml and GLURP-R2 = 3.6 µg/ml) and Gaya (CSP = 7.7 µg/ml and GLURP-R2 = 6.5 µg/ml) (*p *= *0.05* and *p *= *0.05*). The analysis of the difference between the median concentration of anti-CSP IgG and anti-GLURP-R2 in Zinder and Gaya was statistically significant (CSP: *p *= *0.008* and GLURP-R2: *p *= *0.017*).

### Anti-CSP and GLURP-R2 IgG median antibody concentrations by RDT results

The median concentrations of anti-CSP and GLURP-R2 IgG antibodies by RDTs results are in Table 2. When subdividing the groups into those that are RDTs positive or negative and compared to the differences between the median concentrations of antibodies responses against CSP and GLURP-R2 in each groups, no significant difference is observed (*p *= *0.093 and p *= *0.539*).

**Table 2:** Anti-CSP and GLURP-R2 IgG antibody concentrations compared between RDT positive and negative

Antibodies	RDT +	RDT−	*P*
CSP			*0.093*
Median	9.4 (IQR: 29.9)	6.5 (IQR: 16.4)	
GLURP-R2			*0.539*
Median	8.3 (IQR: 35.4)	6.0 (IQR: 20.1)	

RDT +  = Positive rapid diagnostic test and RDT− = Negative rapid diagnostic test. IQR: Interquartile range. Comparison is performed by Mann–Whitney test. The significance limit was p < 0.05

## Discussion

This study demonstrates that seasonal malaria chemoprevention with SPAQ has an effect on blood stage antibody responses and pre-erythrocytic stage of *P. falciparum* infections in Niger. An increased antibody titres are observed in SMC/SPAQ implementation areas. This contradicts hypothesis that SMC/SPAQ could reduce immunity to erythrocyte and liver-stage antigens.

The total IgG antibodies to liver-stage vaccine candidate antigen CSP and blood stage antigen GLURP-R2 are significantly higher in Zinder where SMC has been implemented for 3 years, as opposed to Gaya, where SMC has been implemented for a year, and Dosso, as well, which has never benefitted from SMC. This is consistent with a previous intriguing finding that demonstrated sustained protection during one year of follow-up, in children who have received intermittent preventive malaria treatment [22].

The concentrations of both antibodies against CSP and GLURP-R2 revealed an increase with SMC implementation probably as a result of the decrease of either liver-stage maturation or erythrocyte stage by SPAQ. This, contrasts with other studies [12–14, 23], which found a decrease in the titers of antibodies after SMC delivery. Previous studies established that chemoprophylaxis conferred protective immunity against reinfections when anti-malarial drugs are not present [24–26]. The inhibitory effect of SP on pre erythrocyte stage [27, 28], and AQ on erythrocyte stage was previously described [29]. Friesen J et al. showed in the murine malaria model induction of anti-malarial immunity by pyrimethamine prophylaxis during exposure to sporozoites and attenuation by pyrimethamine permits hepatocyte invasion but appears to block intrahepatocytic replication [24]. The increase in antibodies concentration is believed to be linked to the immune system’s exposure to an attenuate hepatic stage parasites or complete suppression of blood-stage parasites, thereby resulting in an increase of IgG antibody concentration to CSP and GLURPR2 antigens in the sites where SMC was implemented.

Others studies showed that sulfadoxine does not affect liver stages, pyrimethamine has some inhibitory effect on liver stages in *Plasmodium yoelii* models [30], but there are high levels of resistance to pyrimethamine in SMC countries [31]. In Niger. a high prevalence (> 60%) of mutations N51I, C59R and S108N in *Pfdhfr* gene, known to be associated with resistance to pyrimethamine was observed [32]. In vaccine trial cohorts in the Gambia [33], SP did not affect the incidence of low level *P. falciparum* infections detected by PCR, consistent with SP affecting blood stage and not liver stage parasites.

This is the first use of antibody elution from RDT filter paper for the assessment of CSP and GLURPR2 antibody concentration in Niger. As demonstrated by Amrish Baidjoe et al. antibody elution from filter paper is an operationally attractive approach [17]. RDT cassette can be used to monitor molecular markers of malaria drugs resistance and study anti-malarial immunity in Niger. Successful preservation and recovery of intact IgG in these conditions were noted, but further studies should investigate the effect of different storage conditions on sample quality. There was no significant differences between positive and negative RDTs cassettes median concentrations of antibodies responses against CSP and GLURP-R2. This may be due to anti-CSP and anti-GLURP-R2 having long half-lives or possible specificity loss due to undercoated plates.

The comparative analysis of antibodies concentration against CSP and GLURP-R2 antigens between age groups by district, observed no significant differences (CSP: *p *=* 0.6813*; GLURP-R2: *p *=* 0.0760*). No significant differences between positive and negative RDTs were observed, perhaps because of the small number of samples and the short period of observation, which, no doubt, limit the results of this study.

There are other important hurdles too:

- SMC status of all children included in the study was unknown, the sample size is small (from three districts, only two marker of immunity have been measured).
- Differences exist in RDT coverage between sites and collections.
- There is differences in endemicity between Zinder, which is mesoendemic, and Gaya and Dosso, which are hyperendemic. This suggests that differences in transmission intensity may be a major confounder, and future studies should aim to compare areas with the same transmission intensity.
- It is not possible to give the coating of the antigens concentration in order to directly compare them with other studies following the same protocol.
- Different antigens have different coating efficiencies, therefore, coating at the same concentration without optimization may result in a reduction in signal or specificity if over or under-coated, respectively.
- It is not possible to state that the quantity of RDT fragment eluted is the same per sample for antibody titres.
- It is not possible to interpret the immune responses in terms of implications for the risk of malaria. This approach might be useful for monitoring rates of acquisition of immunity in older children who have stopped receiving SMC.

## Conclusion

This data suggest that SMC by SPAQ have an effect on antibody responses against pre-erythrocytic stage and blood-stage antigens. However, other factors that have a significant influence on antibody titers, such as transmission intensity, may confound this. Future studies are necessary to provide a better understanding of the impact of SMC on malaria immunity in Niger. The duration of SMC administration may increase the antibody concentration of *P. falciparum* blood stage antigen GLURP-R2 and pre-erythrocytic stage antigen CSP. RDT filter paper serum elution methodology can significantly reduce the workload and cost in large-scale epidemiological and immunological studies.

## Data Availability

The datasets used and/or analysed during the current study are available from the corresponding author on reasonable request.
